# Superhydrophobic and Compressible Silica-polyHIPE Covalently Bonded Porous Networks via Emulsion Templating for Oil Spill Cleanup and Recovery

**DOI:** 10.1038/s41598-018-34997-1

**Published:** 2018-11-14

**Authors:** D. B. Mahadik, Kyu-Yeon Lee, R. V. Ghorpade, Hyung-Ho Park

**Affiliations:** Department of Materials Science and Engineering, Yonsei University, Seoul, 03722 Korea

## Abstract

We synthesize porous polyHIPE networks with silanol functionalities in the polyHIPE backbone. These silanol functionalities are used for covalent bonding with silica aerogels embedded in the polyHIPE. Covalent bonding between silica and polyHIPE networks are confirmed using Fourier-transform infrared spectroscopy and scanning electron microscopy. Silica aerogels covalently bonded with polyHIPE network show macroporous and mesoporous morphologies and possess excellent properties like high bendability, high elasticity, superhydrophobicity (~160°), low density (~0.128 g/cm^3^), and low thermal conductivity (~0.045 W/m·K). Oil absorption from water/oil mixtures and recovery of the absorbed oil (by squeezing) from flexible silica-polyHIPE networks is studied. The silica-polyHIPE is shown to absorb crude oil ~16-times its own weight and can be reused multiple times after recovery. Hence, such materials are very important for oil spill cleanup applications from aqueous systems.

## Introduction

Silica aerogels have been known for many years and have found applications in oil separation from aqueous media^1^, drug delivery, tissue engineering^2^, catalyst supports, filtration devices^3^, water repellent coatings/materials, etc^4,5^. However, the silica aerogels are flimsy solids with hydrophilic in nature because of the polar surface hydroxyl (−OH) groups^6^. Hydrophobicity of aerogels can be enhanced by many known techniques, such as surface silylation with alkyl non-polar groups^7^. To overcome the fragile nature of aerogels, many attempts have been made, e.g. by applying a polymer coating^8–10^, or cross-linking polymer with the silica network^11,12^; however, this primes to densification of aerogels. Flexible silica aerogels have recently garnered considerable attention for many applications due to their hydrophobic and flexible nature; however, their applications are hindered owing to their fragile nature^13–15^. Flexible silica aerogels obtained from the methyltrimethoxysilane (MTMS) precursor are widely studied for oil spill cleanup application because they absorb ~15-times their own weight of oil^16,17^. However, oil recovery by mechanical force is difficult because the aerogels break easily to form powders^1^. In our earlier paper^14^, we have reported a silica-polyHIPE composite, prepared by physically embedding flexible silica aerogels (without any chemical bond) in polyHIPE (polymerization by high internal phase emulsion) network scaffolds, which have large void with interconnecting windows. Since both polyHIPE and silica aerogels are flexible and embedded with each other at the micron level, the composite showed good mechanical properties while maintaining the flexible nature. However, with time, after applying multiple compressions and releases, silica aerogels were converted to powder/dust, which decreased the performance of the material. To avoid the dust formation due to physically embedded silica aerogels and most importantly increase reusability of the material, we proposed to develop a strong chemical bond between silica and polyHIPE. Therefore, herein we proposed and executed a novel method for making composites of silica aerogels covalently bonded with polyHIPE while maintaining the flexible nature of the aerogels, and these composites have been studied for oil spill cleanup applications. Though difference in only covalent bond but it matters a lot at actual application of the material, hence we feel this study have ability to resolve oil spill cleanup problem.

**Figure 1:**
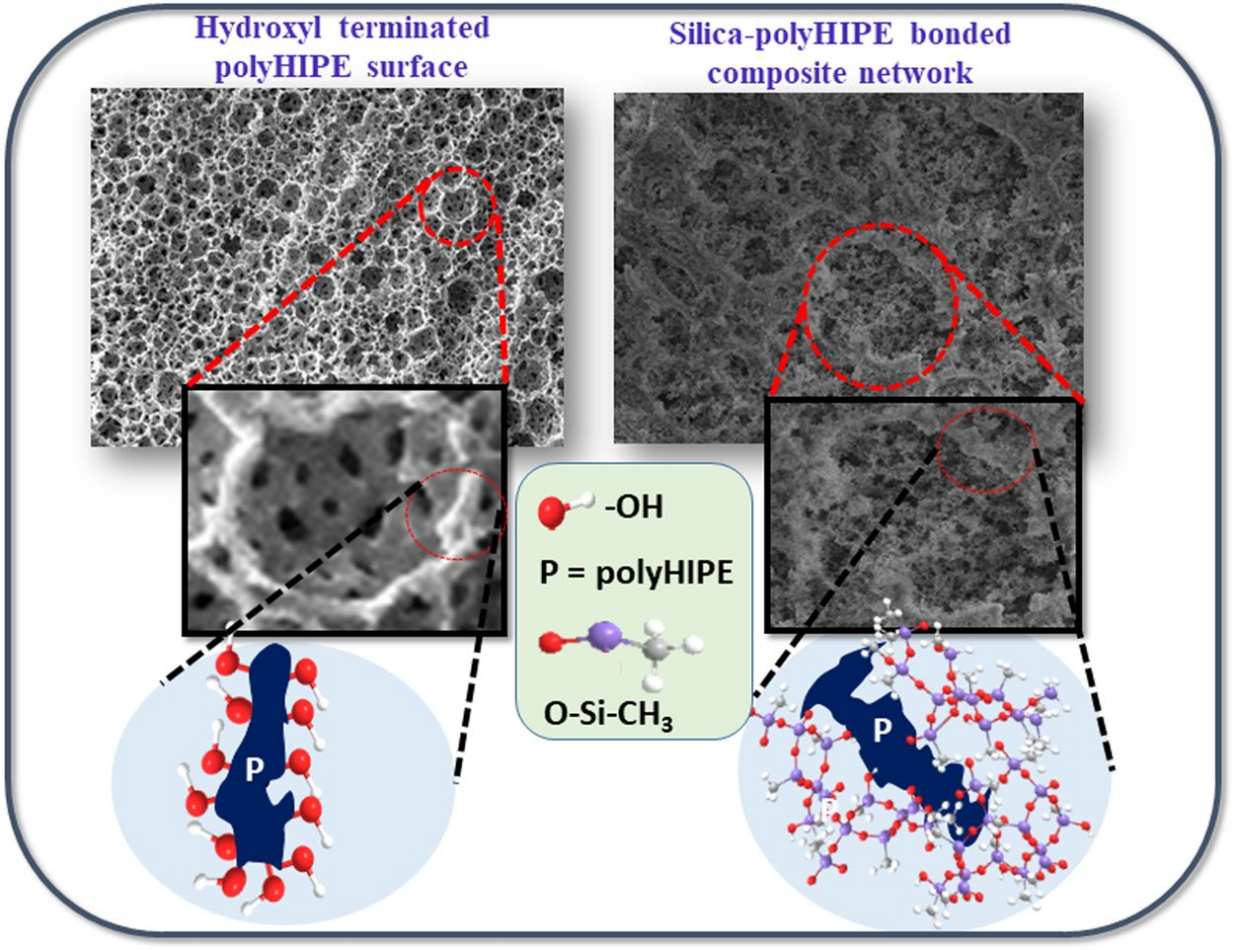
Schematic of hydroxyl terminated polyHIPE and covalently bonded silica-polyHIPE networks.

PolyHIPEs gain huge attention because simple preparation method (HIPE) and this method can be extendable for the synthesis of large size monoliths with well-demarcated porosities^18^. PolyHIPEs are synthesized within high internal phase emulsions (HIPEs) with more than 74% porosity, using free radical polymerization (FRP) of monomers of polymers in surfactant-stabilized water-in-oil (W/O) HIPEs^19^; monolithic porous foams are suitable for several applications under concern. Various functionalities can be incorporated into the porous polyHIPE network by copolymerization^20^. Taking advantage of this, we synthesized porous polyHIPE networks with silanol functionalities in the polymer backbone, which have been used for covalent bonding with the porous hydrophobic silica network embedded in polyHIPE. In this paper, we describe the simultaneous synthesis of a siloxane-functionalized polymer network within flexible polyHIPE and MTMS-based aerogel network. The elastic silica network was reinforced by the elastic macro-porous polymer at micron level via covalent bonding between them. The schematic presentation of synthesis of hydroxyl terminated polyHIPE and covalently bonded silica-polyHIPE networks as shown in the Fig. 1. The synthesized composite materials showed high flexibility, high elasticity, superhydrophobicity, and low thermal conductivity.

## Results and Discussion

### Properties of Silica-polyHIPE covalently bonded monoliths

**Figure 2:**
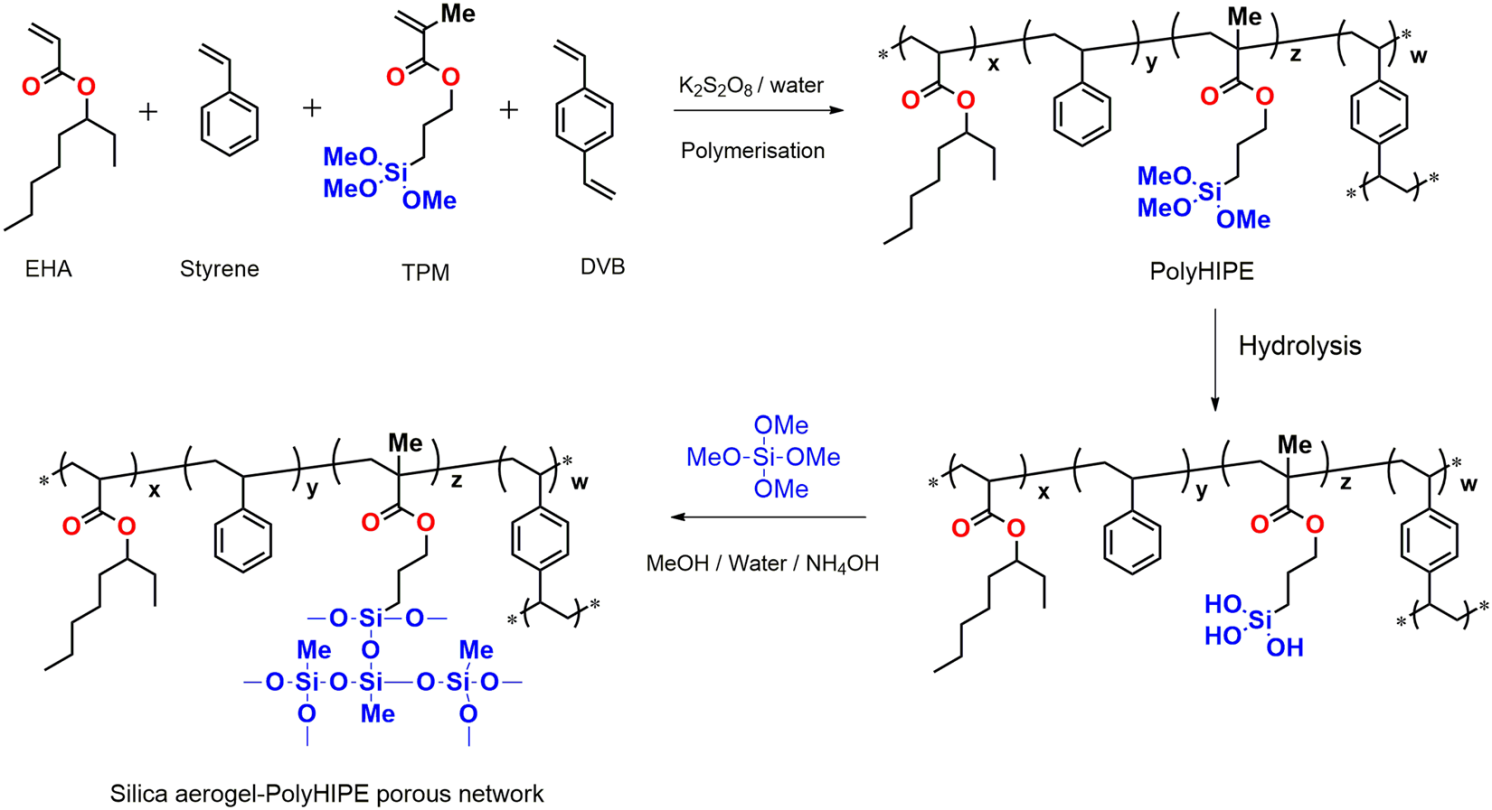
Synthesis of silica-polyHIPE covalently bonded porous networks.

**Figure 3:**
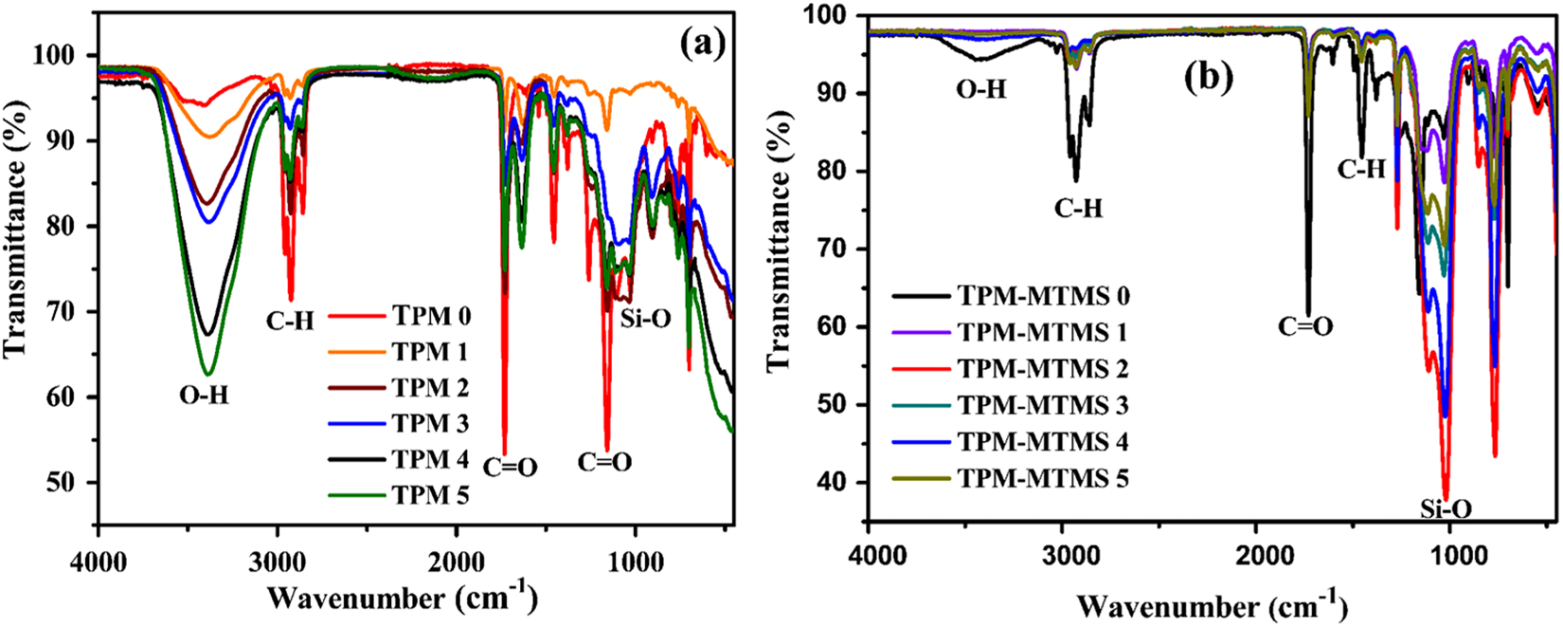
FTIR spectra of (**a**) TPM-polyHIPE networks with increasing TPM contents and (**b**) silica-polyHIPE bonded networks.

A water-in-oil (w/o) HIPE template was used for the preparation of polyHIPEs from the monomers TPM, EHA, styrene and DVB stabilized by Span 80. A number of porous polyHIPE materials with hydroxyl bond on the surface were prepared with varying TPM amount relative to the total monomer content (Table 1). The designation of samples is given as TPM0,1,2,3,4 and 5 with increased content of TPM monomer as given in Table 1. The monomers were incorporated in the continuous phase, while the aqueous phase is used to pattern the porosity. Crosslink densities of all polyHIPEs were kept constant by addition of 25 mol% of DVB with respect to other comonomers. The water-soluble free radical initiator (potassium persulfate) used to yield solid porous monoliths after polymerization^21,22^. All the polyHIPE monoliths show interconnected open porous architectures with bimodal pore size distributions. A combination of styrene and EHA monomers was selected because they have been used to prepare flexible polyHIPEs, and TPM was selected for covalent bonding between the porous polymer network and the silica aerogel. Polymerization of EHA, TPM, and DVB (Reaction mechanism Fig. 2) forms a network containing methoxy groups at chain end. These methoxy groups were hydrolyzed to form hydroxyl (-OH) groups, as confirmed by an FTIR analysis of polyHIPE prepared without TPM and with increasing TPM contents (Fig. 3(a)). The structure of the polyHIPE foams are in agreement with the polymer structure, as expected. The polyHIPE networks prepared without TPM and with increasing TPM contents are clearly distinguishable based on their FTIR spectra. The intensity of the peak at 3450 cm^−1^ (attributed to the −OH bond) increased with increasing TPM content. This confirms that the amount of -OH groups in polyHIPE increased with increasing TPM content. These surface -OH groups are highly favorable for bonding with silica, as shown in the Fig. 2. The small peak at ~1080 cm^−1^ in the FTIR spectra of all samples except TPM0 indicates the presence of Si-O-Si, confirming the TPM bonding with polymerization of polyHIPE monomers^19,23^. The sharp peaks at 1155 and 1730 cm^−1^ in all spectra (Fig. 3(a)) were attributed to the carbonyl function group (C=O bond). The peak at 2925 cm^−1^ indicates the presence of C-H bonding in the polyHIPE materials^24^. After preparation of hydroxyl-terminated polyHIPE monolith, the material was further soaked in hydrolyzed MTMS sol to allow gelation of silica network in the polyHIPE scaffold and was subjected to supercritical drying. The bonding between silica aerogels network and polyHIPE was analyzed using FTIR spectroscopy (Fig. 3(b)). The peak at 3450 cm^−1^ (attributed to the −OH bond) disappeared for all samples except TPM0, indicating that the hydrolyzed MTMS forms covalent bond with polyHIPE with condensation of water. The intensity of the peaks at around 1450 and 2900 cm^−1^ (Fig. 3(b)) increased with increasing TPM content, corresponding to the increased methyl group contents in the porous networks. This enhances the hydrophobic nature of the composite aerogels because contact angle increased with increasing TPM content (Table 2 and Fig. S1). The strong absorption peak observed in all spectra at 1080 cm^−1^ indicates the formation of Si-O-Si network, and the intensity of this peak increased after MTMS modification of polyHIPE. FTIR analysis confirms that TPM produces hydroxyl bonds on the polyHIPE surface and covalent bonds with polyHIPE are formed upon addition of hydrolyzed MTMS, as shown in reaction mechanism.

**Table 1:** Composition of the silica-polyHIPE samples.

Designation	Styrene (g)	DVB (g)	EHA (g)	TMP (g)
TMP 0	1.0	1.25	3.54	0
TMP 1	1.0	1.45	3.54	1.19
TMP 2	1.0	1.66	3.54	2.38
TMP 3	1.0	1.87	3.54	3.57
TMP 4	1.0	2.08	3.54	4.76
TMP 5	1.0	2.50	3.54	7.15

**Table 2:** Physical properties of the silica-polyHIPE bonded networks.

Designation	Density (g/cm^3^)	Thermal conductivity (W/m·K)	Water contact angle (°)	BET surface area (m^2^/g)	Flexible nature
TMP 0	0.120	0.1084	0	12	Highly flexible
TMP 1	0.128	0.0450	160	115	Flexible
TMP 2	0.130	0.0442	162	270	Slightly flexible
TMP 3	0.136	0.0455	163	350	Hard
TMP 4	0.139	0.0471	163	401	Monolith not formed
TMP 5	0.142	0.0492	165	468	Powder form

The physical properties of silica-polyHIPE covalently bonded samples are shown in Table 2. The sample TPM0 is a polymer of DVB, EHA, and styrene monomers, which shows flexibility and hydrophilicity. The density of the samples increased from 0.120 to 0.142 g/cm^3^ as silica content is increased with increasing TPM content. Thermal conductivities of the silica-polyHIPE samples were lower than the TPM0 sample because microporous MTMS-based silica aerogels grown in the macrovoids of polyHIPE scaffold reduced thermal conduction. The water contact angle was measured for quantifying the hydrophobicity of the samples; the MTMS-modified polyHIPE showed that all the samples were superhydrophobic in nature. The composite of polyHIPE with MTMS silica aerogels shows water contact angle in the range 160–165°. Contact angle goniometer pictures as shown in Fig. S1. All samples showed the least amount of contact angle hysteresis (<12°), TPM1, 2, 3, 4 and 5 shows CAH 12°, 8°, 9°, 6° and 5°, respectively, indicates small sliding angle.

The addition of TPM commonly enhances the hardness of the samples; TPM1 and TPM2 samples showed flexible nature while the rest of the samples showed hard nature. The textural properties of silica-polyHIPE covalently bonded aerogel materials were measured using Brunauer-Emmett-Teller (BET) analysis and the surface area values are shown in Table 2. It is well known that, since polyHIPE (TPM0) has very low specific surface area (12 m^2^/g) though porosity is high due to it possess an open macro-porous morphology with pore size (10–20 µm)^25^. The adsorption and desorption isotherms for the samples TPM1, 2, 3, 4 and 5 are shown in Fig. S2. This indicates that the TPM1-5 samples have similar isotherms with increased adsorption volume. The adsorption and desorption curves for the TPM1, 2, 3, 4, and 5 samples show the characteristic type IV shape with H1 hysteresis loop, as per the IUPAC classification scheme^14^. This type of hysteresis loops indicates the capillary condensation of N_2_ molecules in the pores with size of 2–50 nm, suggests that aerogels are mesoporous in nature. As flexible MTMS-based silica aerogel possesses mesoporous morphology. Therefore, silica-polyHIPE composite material shows similar isotherms as MTMS aerogels with less adsorption volume of N_2_. The BET surface areas of TPM 2, 3, 4, and 5 samples were 270, 350, 401, and 468 m^2^/g, respectively. Hence, BET analysis suggests that the amount of silica network in the polyHIPE network increases with increasing TPM content. Therefore, surface area should increase with an increase in the percentage of silica network because it possesses mesoporous structure. Thermal conductivities of all the samples were measured by C-T meter and the values are given in Table 2. The thermal conductivity of TPM0 (0.1084 W/m·K) is larger than all other samples containing silica network (~0.045 W/m·K) because the silica aerogels possess low thermal conductivity. Therefore, such flexible, water-repellent, lightweight materials with thermal insulation will be highly applicable for thermal insulation of buildings and the flexible nature of the material will allow it to cover any shape^26^.

### Microstructural analyses

**Figure 4:**
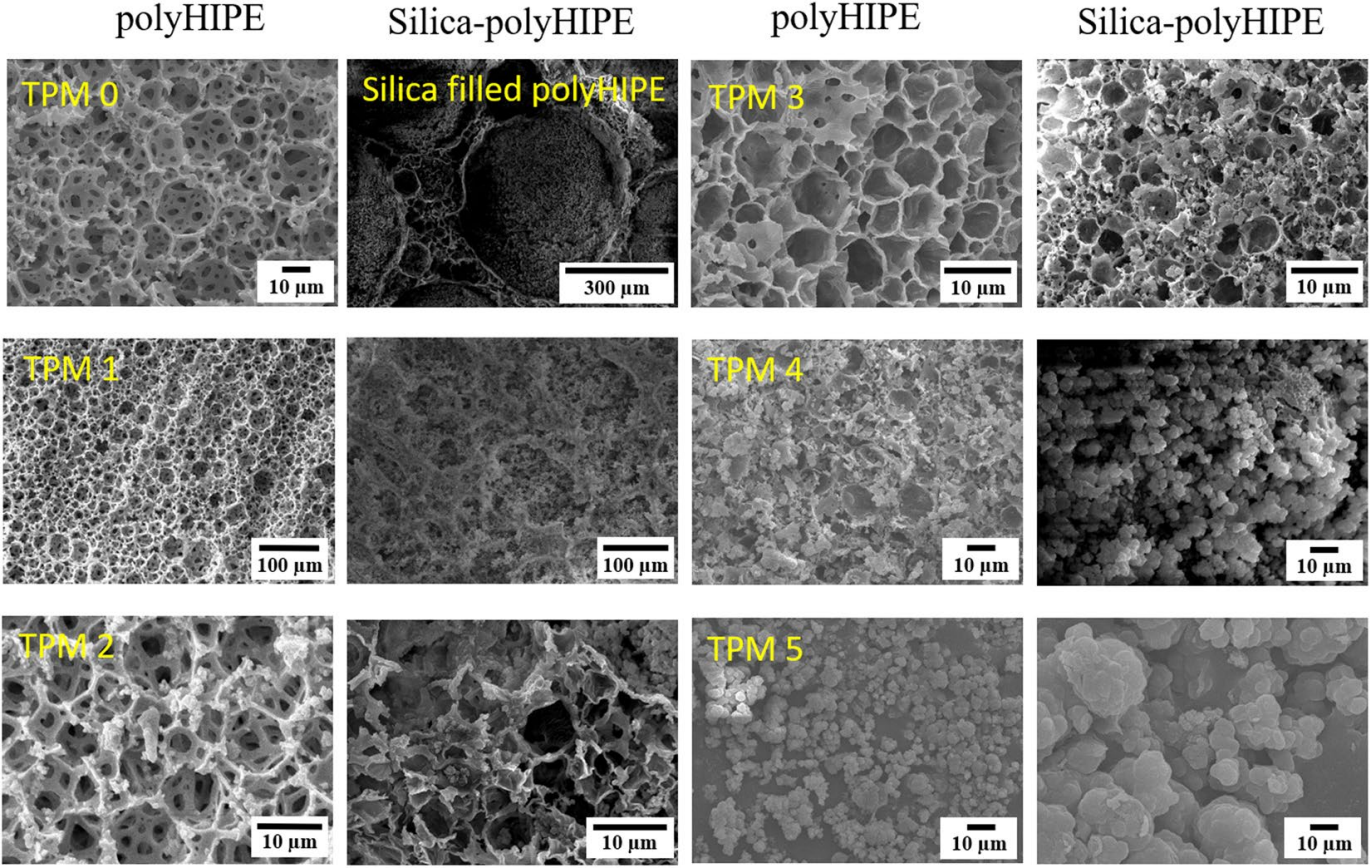
SEM images of polyHIPE networks with increasing TPM contents and the corresponding sample after silica-polyHIPE bonding for TPM0-5.

The surface morphologies of silica-aerogel-embedded polyHIPE materials with increasing TPM content were analyzed using scanning electron microscopy (SEM) and the micrographs are shown Fig. 4. Basically, polyHIPE materials are emulsion-derived foams possessing open cellular morphology obtained by free radical polymerization. The morphology of the polyHIPEs depends on the surfactant because it plays a crucial role in emulsion sol stabilization, and also on the amount of free radical initiator used to ease the polymerization process^18^. The SEM images of the polyHIPE samples TPM0–TPM5 (Fig. 4) shows open-cellular morphologies with macroporous voids having interconnecting windows in polyHIPE. This can be clearly seen in the SEM images of TPM0 and TPM1. The lower-magnification SEM image of the samples shows polyHIPE network filled with the silica aerogel network. The surfactant concentration plays a crucial character during sol stabilization in forming uniform/disorderly open- or closed-pore morphology during EHA-styrene/DVB polyHIPEs polymerization. With increasing TPM content, polyHIPE morphology was distorted and for TPM 3–5, the pores became uneven. For TPM 2, the interconnecting windows disappeared and the polymer network appeared thicker than TPM0. The SEM image of the TPM0 sample depicts spherical voids (~15 μm) with interconnected windows (1–2 μm) and hollow spaces with open pore cell morphology. However, MTMS based silica aerogels show an open porous morphology with different sizes and compact morphologies (around 10 times smaller) compared to polyHIPEs. The SEM image for TPM1 after silica modification shows that small clusters of silica aerogels initiated polymerization with polyHIPE. For TPM2, there is a slight increase in the silica network content and increased silica clusters are observed for increased TPM contents. FTIR analysis suggests that hydroxyl groups increased with increasing TPM concentration, which enhances the growth of silica clusters; hence, SEM results are in good agreement with FTIR results and the proposed reaction mechanism shown in Fig. 2. Hence, micrographs of TPM1-4 show that the polyHIPE network appears as a scaffold for the silica network and silica network is grown/coated in the polyHIPE matrix. This innovative way of preparing covalently bonded silica-polymer composites allows the filling/coating of macron-sized open pores of polymer matrix with small-pore-size silica matrix, as perceived from the SEM micrographs. Previous reports have stated that silica nanoparticles are used in emulsion stabilization and the presence of silanes effectively alters the morphology in voids and the size of the voids^14,27^, because functionalization of silica nanoparticles with silanes affects the locus of beginning (organic phase interface) and the mechanism of free radical polymerization. Generally, hexagonal voids are observed for polyHIPEs synthesized by FRP, as seen for TPM0. However, the polyHIPEs with increased TPM contents, i.e. TPM1-5 shows polyhedral voids with closed interconnected windows. This morphology is analogous with the porous structures of silane-modified nanoparticles or Pickering HIPEs that were affected by the silane reaction mechanism^27^. Hence, the polyhedral morphology is observed for higher silanol contents due to the polyhedral voids produced by interfacial FRP initiation^28^.

### Thermal stability analysis

Thermal stabilities of covalently bonded silica-polyHIPE composite samples in air were analyzed using thermogravimetry (TG). TG profiles of all the samples (Fig. S3) showed sharp weight loss starting at 307 °C and continuing up to 464 °C, thus confirming the decomposition of the organic material^12,29^. The decomposition of organic materials from MTMS-based silica aerogels and polyHIPE porous networks is well known and confirms the thermal stability around 280 °C, as reported earlier^10^. Here silica and polyHIPE are covalently bonded with each other, which might lead to the enhanced thermal stability of composite materials from 280 to 307 °C in air. The TG profiles of the samples also show that weight loss of samples the TPM0, 1, 2, 3, 4, and 5 are 2%, 10%, 14%, 16%, 18%, and 41%, respectively. Thus, the loading amount of silica in the polyHIPE network can be tuned over a wide range.

### Mechanical properties

**Figure 5:**
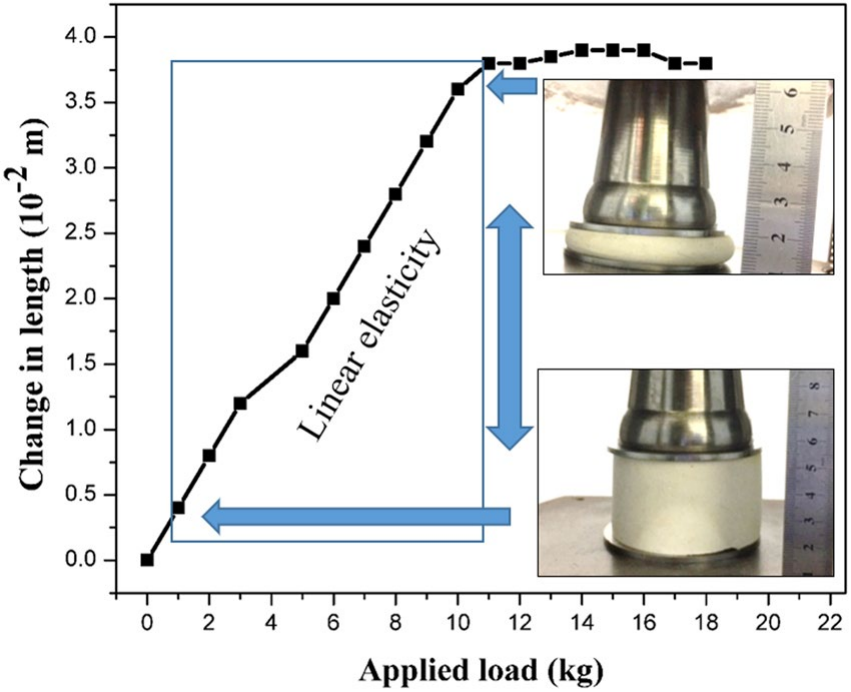
Plot of the applied load (in kg) versus change in length of silica-polyHIPE sample (TPM0).

To determine material parameters of the silica-polyHIPE flexible foams as function of test parameters (diameter of specimens and strain), a number of compression tests were executed. To obtain the mechanical properties of the TPM1 sample (fully elastic deformation), 5 compression tests have been accomplished at the MTS 810 Elastomer Test System. In this test, the silica-bonded polyHIPE composite cylindrical sample with diameter of 100 mm and height of 50 mm was placed between two flat cylindrical stainless steel plates. Then a compressive force was applied at a crosshead displacement at the rate of 2.5 mm/min for each 25.4 mm of sample thickness. Consequently, the compression of sample was characterized by measuring applied force versus the corresponding change in length (*ΔL*, *L* being the original length of the aerogel) *ΔL* was plotted against the mass applied (*m*), and the slope (*ΔL/m*) of the graph was measured for the Young’s modulus measurement using following equation;

$$\mathrm{Young}\mbox{'}s\,{\rm{modulus}}\,(Y)=mgL/\pi \,{r}^{2}{\Delta }L=(Lg/\pi \,{r}^{2})/slope$$

where *g* is the acceleration due to gravity and *r* is the radius of the silica-polyHIPE sample^13^. The region of elasticity is initially linear; however, after a load of 11 kg is applied, it obtains a downward concave shape, followed (at a strain of ~24%) by a straight elastic collapse plateau. After compression to ~76% of its original shape, the silica-polyHIPE specimen will (almost) fully return to its original shape; therefore, the deformation is fully elastic, as observed from the photographs and plot shown in Fig. 5. Further, the stress-strain response clearly shows a material-dependent behavior. The Young’s modulus was calculated to be 10.47 MPa from the slope measured from Fig. 5 and using the above mentioned equation. The obtained Young’s modulus signifies significant elastic behavior compared with that reported for similar compressible foams^30,31^. Compression can be done up to 76% of its original volume because it regains its original shape within 30 s after release. Hence, such material will be highly useful for oil recovery due to its good mechanical properties.

### Oil absorption and recovery studies

**Figure 6:**
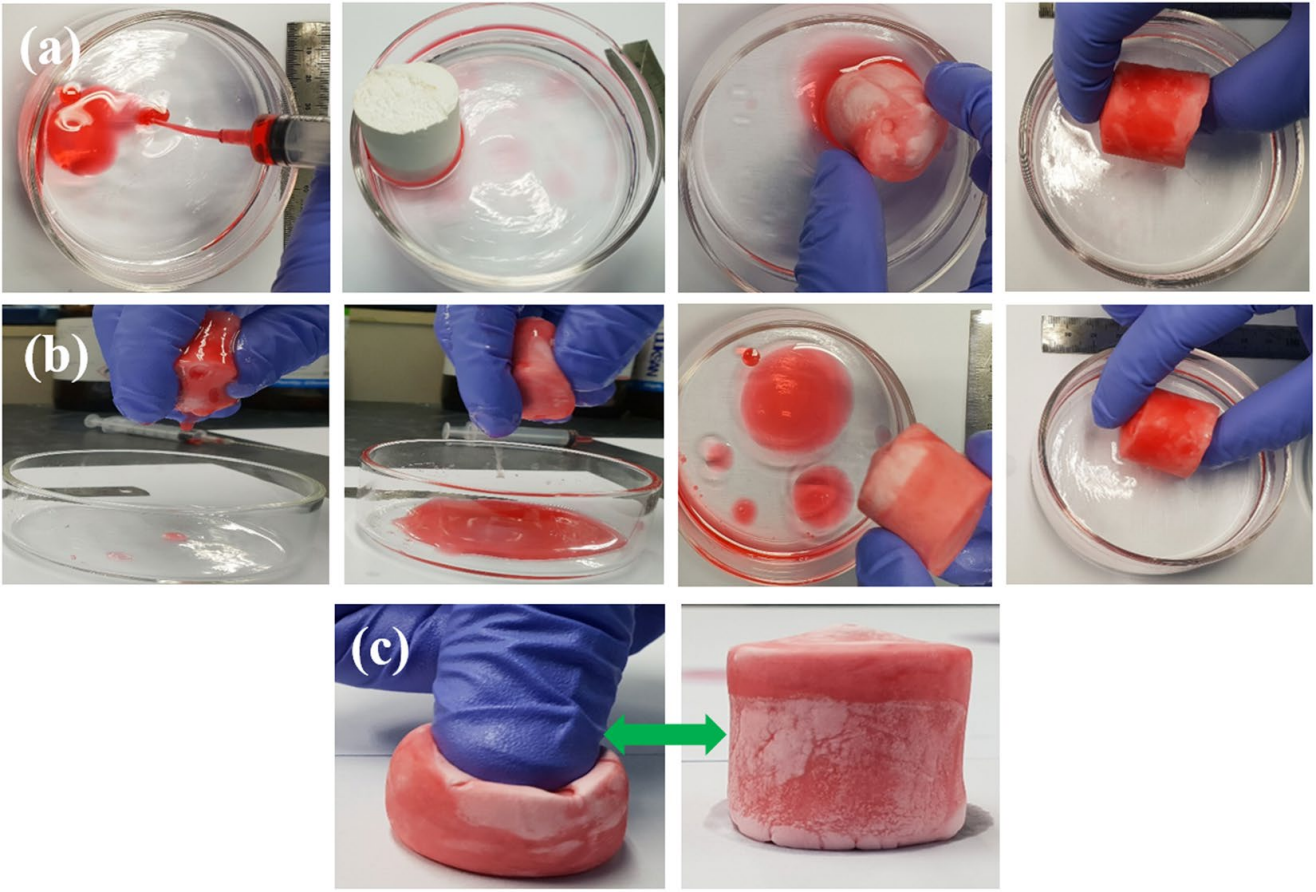
(**a**) Static images taken from a repetitive sorption/compression/re-sorption cycle on the surface of a water bath and crude oil. (**b**) Images correspond to recovery of oil by compression and again re-sorption of oil from oil-water mixture. (**c**) Images shows compression of sample and after release it regain original shape and size.

Low-density open-cellular sponge of different materials are known for oil absorption from water-and-oil mixtures^32^. Most reported studies have shown oil absorption properties of materials; however, there have been few studies on oil recovery from sponge. Barry *et al*. synthesized a polyurethane or polyimide polymeric material foam infused with inorganic (Al_2_O_3_) material using sequential infiltration synthesis based functionalization scheme and in a subsequent step, a silane agent is grafted to the metal oxide coating, rendering the oleophilic nature (oil sorption capacity ~30–90 g per 1 g)^33^. Results shown that these functionalised foams can absorb seawater (~30 g) indicates hydrophilic nature. Also scalable production with uniform coating of metal oxide on large size foam looks challenging as it involves a synthesis method which is similar to atomic layer deposition technique. Since most materials are highly porous, hard, and brittle, oil recovery is difficult and very slow. Therefore, materials with high flexibility, hydrophobicity, and porosity will be highly suitable for absorption and recovery of oil. Since the silica-polyHIPE material (TPM1) reported herein has suitable characteristics, an oil absorption and recovery study was carried out. The outer surface of the material is oil-absorbing (silica aerogels) and water-repellent, while the inner polyHIPE network is highly porous with interconnecting windows with large neighboring voids (~20 µm in size) where oil can be stored for a short period. Crude oil and water mixture were taken in a petridish and sorption/de-seorption static pictures are shown in the Fig. 6. The sample weight was noted after oil absorption; then, sample TPM 1 was compressed for oil recovery, released, and weighed to compare the weight of the sample before oil absorption and after oil recovery. 1 g of TPM1 sample absorbed 18 g oil and after recovery, 16 g oil was obtained in the first cycle. The 2 g loss of oil may be because oil absorption leads to wetting of the silica-polyHIPE network. The sample reusability measurements were done by repeating same sample for sorption and de-sorption of crude oil. This cycle of sorption/de-sorption of crude oil from water was repeated for 25 times and the absorbed mass of crude oil per cycle is shown in Fig. S4. The measurements show nearly same sorption capacity from cycle 2 to 25 cycles; indicates the materials have good cycling stability. Also measured absorption capacity of the silica-polyHIPE (TPM1) sample for various organic liquids. Various organic liquids such as pentane, hexane, heptane, octane, toulene, methanol, ethanol, petrol and crude oil were used for sorption/de-sorption analysis. The mass of various organic liquid absorbed by the silica-polyHIPE composite (TPM1) material are shown in Fig. S5. The sorption capacity of the material is observed in the range ~8 to 18 g of liquid per 1 g of sample. Silica network bonded with polyHIPE has increased strength and maintains its characteristic properties even after a number of compression and release tests. Hence, the proposed reaction mechanism is proven highly effective from an application point of view because the material maintains its properties due to covalent bonding as shown in the supplementary video. For other samples TPM2, 3, 4, and 5, the amounts of crude oil absorption are 18, 20, 21, and 24 g, respectively. However, due to the hard nature of the samples, immediate oil recovery by force is difficult. Therefore, such multifunctional silica-polyHIPE (TPM1) material will be very important for oil absorption and recovery in oil spills accidents^34^.

## Conclusions

Flexible covalently bonded monolith porous networks of silica-polyHIPE were prepared by emulsion templating followed by a sol-gel process. FTIR analysis confirms the formation of hydroxyl groups on the surface of polyHIPE after addition of TPM to the polyHIPE monomers. Surface modification of the hydroxyl-terminated polyHIPEs was performed using MTMS-based silica aerogels by a sol-gel process. Covalently bonded silica-polyHIPE networks have low density, thermal conductivity, and superhydrophobicity, while maintaining high flexibility. SEM images confirm that the morphologies change from hexagonal to polyhedral upon increasing the TPM content due to silanol Pickering polyHIPEs process. Thermal analysis shows that silica content increased up to 41% upon increasing TPM content. The Young’s modulus of silica-polyHIPE flexible sample (TPM1) is calculated to be 10.47 MPa. The crude oil sorption/de-sorption capacity of the material is ~16 g of liquid per 1 g of sample. The cycle of sorption/de-sorption of crude oil from water was repeated for 25 times with nearly same sorption capacity; indicates the materials have good cycling stability. High flexibility and covalent bond between polyHIPE and silica network avoid dust formation and reusability without losing its sorption capacity. Also material shows low thermal conductivity and has good thermal stability (~300 °C) in air atmosphere and superhydrophobicity (160°). These multifunctional extreme characteristics of polyHIPE-silica composites would be all of interest for applicaion in oil spill clean-up and thermal insulation.

## Experimental

### Materials

MTMS (Sigma), divinylbenzene (DVB), styrene, (Aldrich), (±)-2-ethylhexyl acrylate (EHA) (Aldrich), 3-(trimethoxysilyl)propyl methacrylate (TPM) (Sigma), sorbitanmono-oleate (Span80) (Aldrich), potassium persulfate, calcium chloride hexahydrate (Aldrich), methyl alcohol, oxalic acid, and aqueous ammonia were used without further purification.

### Characterizations

Fourier-transform infrared (FTIR) analysis of silica-polyHIPE aerogels were performed using a Perkin Elmer (USA) IR spectrophotometer. A small slice of sample kept on the sample holder and IR measurement was carried out in 400–4000 cm^−1^ range. The textural properties and morphologies of silica-polyHIPEs was analyzed using a multi-point Brunauer–Emmett–Teller (BET) surface analyzer (TriStar 3000 V6.05 A) and a field-emission scanning electron microscope (FE-SEM, JEOL, Japan), respectively. The thermal conductivities of samples were measured using a C-T meter (Teleph Company, France). Hydrophobicity of samples was quantified using a water contact angle (θ) meter (rame-hart USA), the static contact angle was measured by keeping ~5 µL water droplet on the sample using a microsyringe. For samples having θ > 150^o^, the dynamic contact angles were also measured. The difference between advancing contact angle (θ_A_) and receding contact angle (θ_R_) gives the value of contact angle hysteresis (CAH). The thermogravimetric (TG) studies were performed by a Thermal Analyzer unit (DuPont 9900), under air atmosphere at a heating rate of 5 °C min^−1^. All silica-polyHIPE samples dehydrated in an oven at 100 °C for 1 h prior to thermal analysis. Mechanical compression tests have been performed at the Elastomer Test System.

### Experimental procedure of silica-polyHIPE covalently bonded networks

A series of polyHIPE networks were prepared, with increasing TPM contents, relative to the total amount of organic monomers (10%) and 90% aqueous solution was added drop-wise to obtain a stable emulsion of high-internal-phase i.e. water-in-oil (W/O). The detailed experimental procedure for silica and polyHIPE is followed as per our recently published paper^14^. In brief, styrene, EHA, DVB, and Span 80 were placed in a cylindrical Teflon mold (have lids at both sides). Varying amounts of TPM (0–7.15 g) were added to this monomer solution (Table 1). Then a solution of calcium chloride hexahydrate (2.0 g) and potassium persulfate (0.2 g) dissolved in deionized water (90 mL) was added dropwise at 500 rpm at room temperature in the above monomer solution. The stable emulsion was obtained and allowed polymerization at 65 °C for 48 h. Then the surfactant was removed by washing polyHIPE with water for several times and finally monolith was dried in vacuum at 80 °C for 24 h.

MTMS Silica sol for flexible silica aerogels was prepared by two step sol-gel process^16^. The molar ratio of MeOH/MTMS (M) and H_2_O/MTMS was kept constant at 34 and 8, respectively. The molar concentrations of oxalic acid and NH_4_OH catalyst were fixed at 0.01 M and 10 M, respectively. The base catalyst was added dropwise in the pre-hydrolyzed MTMS silica sol under stirring. Then, silica sol inserted in the polyHIPE monolith using a vacuum oven at room temperature and further kept as it is for gelation. For strengthening of silica network, gel was aged at room temperature for 2 days. Finally, this silica-polyHIPE gel was dried using a methanol supercritical drying system (Parr autoclave, USA).

## Electronic supplementary material

Supplementary Information

Absorbed Oil Recovery of Silica-ployHIPE Composite Aerogel
