# Supplementary material for: Superhydrophobic and Compressible Silica-polyHIPE Covalently Bonded Porous Networks via Emulsion Templating for Oil Spill Cleanup and Recovery
Source: Sci Rep. 2018 Nov 14;8:16783. doi: 10.1038/s41598-018-34997-1 (PMC6235919; doi:10.1038/s41598-018-34997-1)
Supplement: Supplementary file 1 — Supplementary Information [file 41598_2018_34997_MOESM1_ESM.pdf]

Supporting information

Superhydrophobic and Compressible Silica-polyHIPE Covalently Bonded Porous Networks via Emulsion Templating for Oil Spill Cleanup and Recovery

D.B. Mahadik†*, *Kyu-Yeon Lee* †, *R.V. Ghorpade, Hyung-Ho Park**

Department of Materials Science and Engineering, Yonsei University, Seoul 03722, Korea

Corresponding Author: Prof. Hyung-Ho Park

Department of Materials Science and Engineering, Yonsei University, Seoul 03722, Korea

***E-mail:** hhpark@yonsei.ac.kr

Telephone: 02-2123-2853

Co-Corresponding Author: Dr. Dinesh B. Mahadik

***E-mail:** mahadikdinesh7171@gmail.com

† These authors contributed equally to this work.

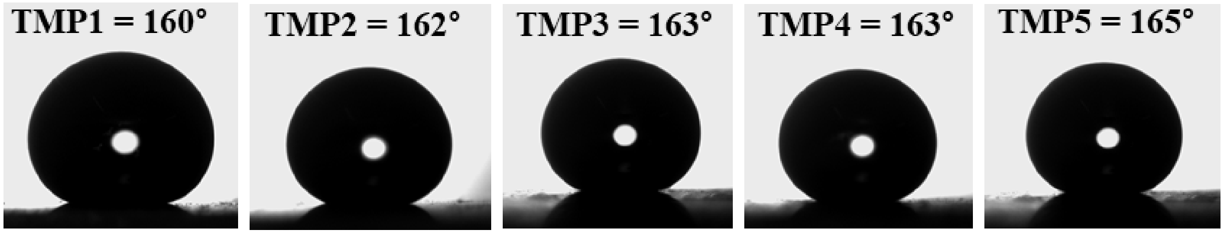

Figure S1. Water contact angle images on silica-bonded polyHIPE materials TPM1, 2, 3, 4, and 5.

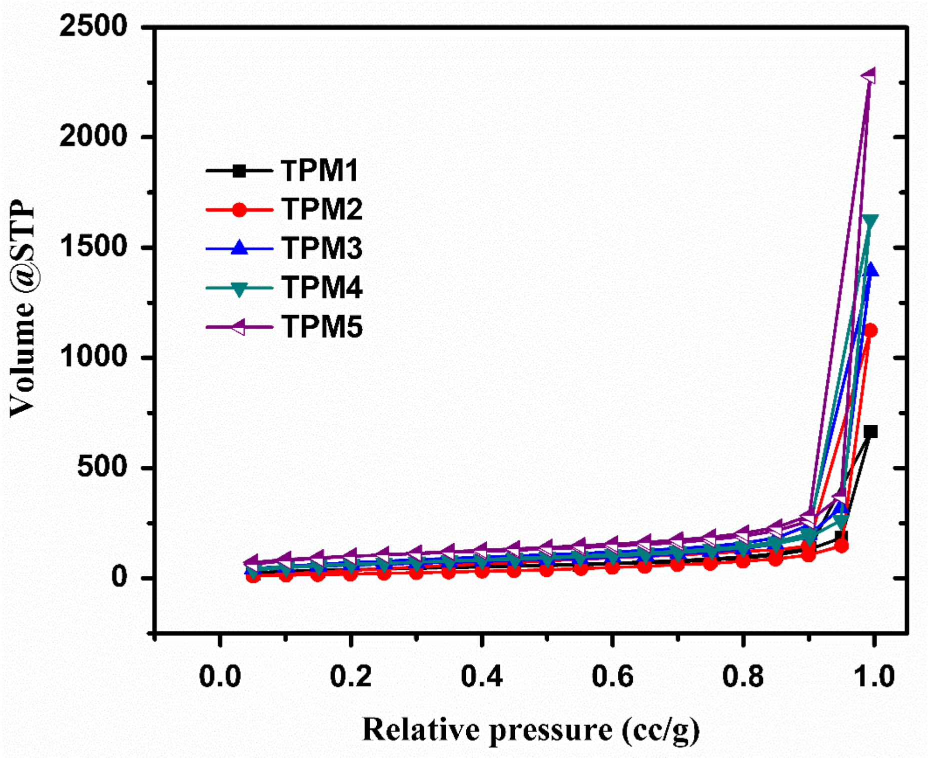

Figure S2. BET adsorption and desorption profiles of sample TPM1,2,3,4 and 5.

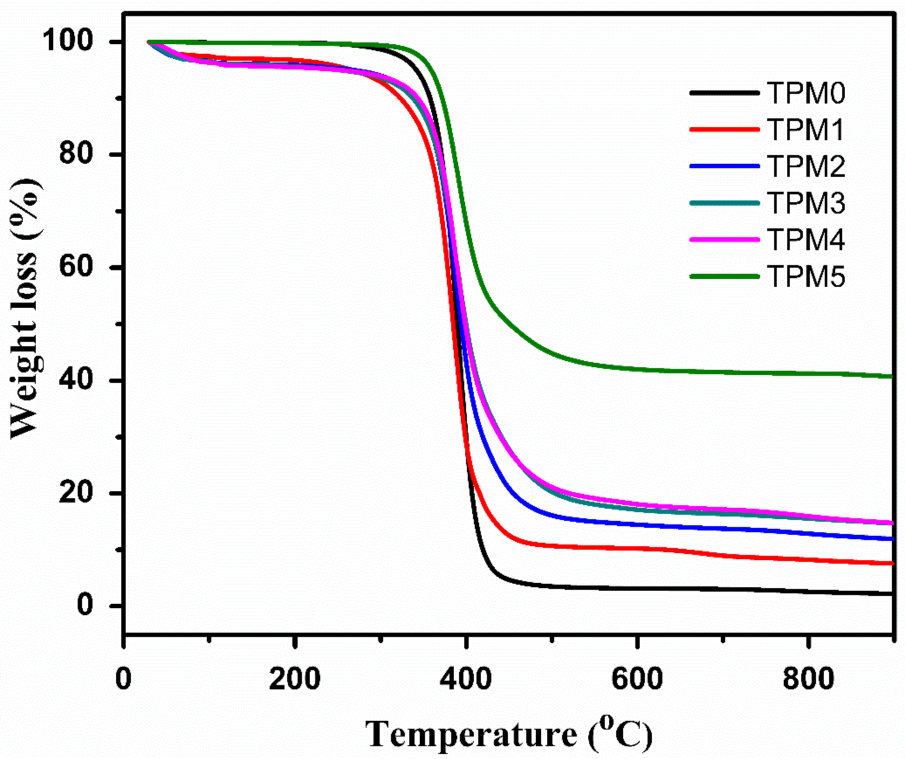

Figure S3. TG profiles of silica-bonded polyHIPE materials TPM0, 1, 2, 3, 4, and 5 in air.

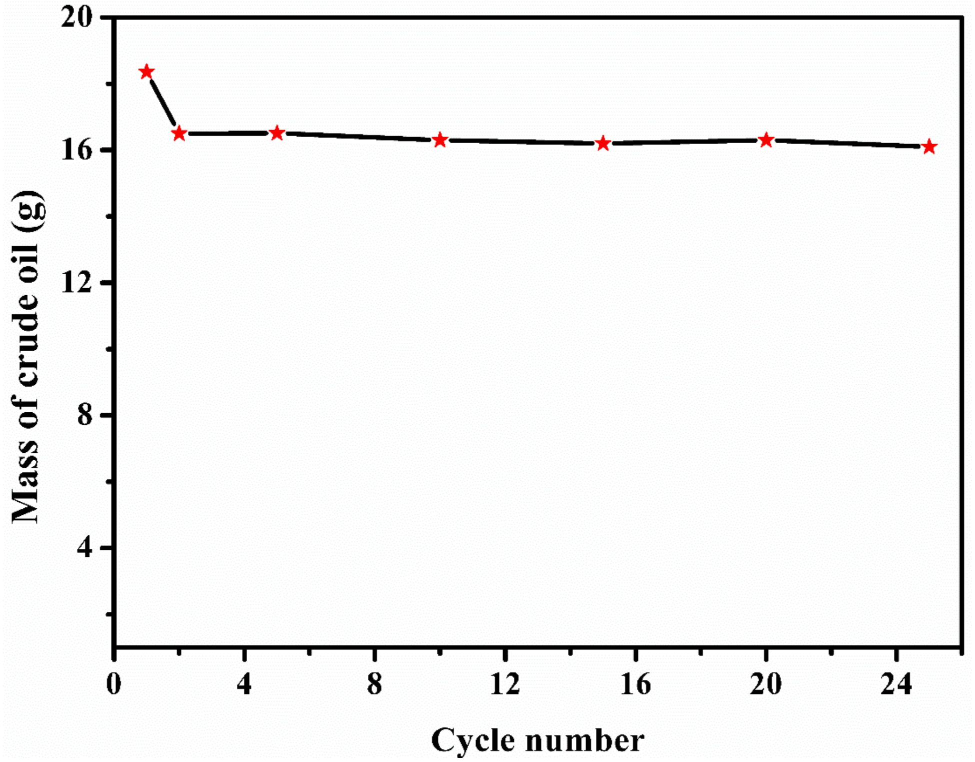

Figure S4. Reusability - Oil sorption capacity of crude oil pump oil following multiple cycles of compression and re-sorption, demonstrating reusability properties of polyHIPE-silica composite material (TPM1).

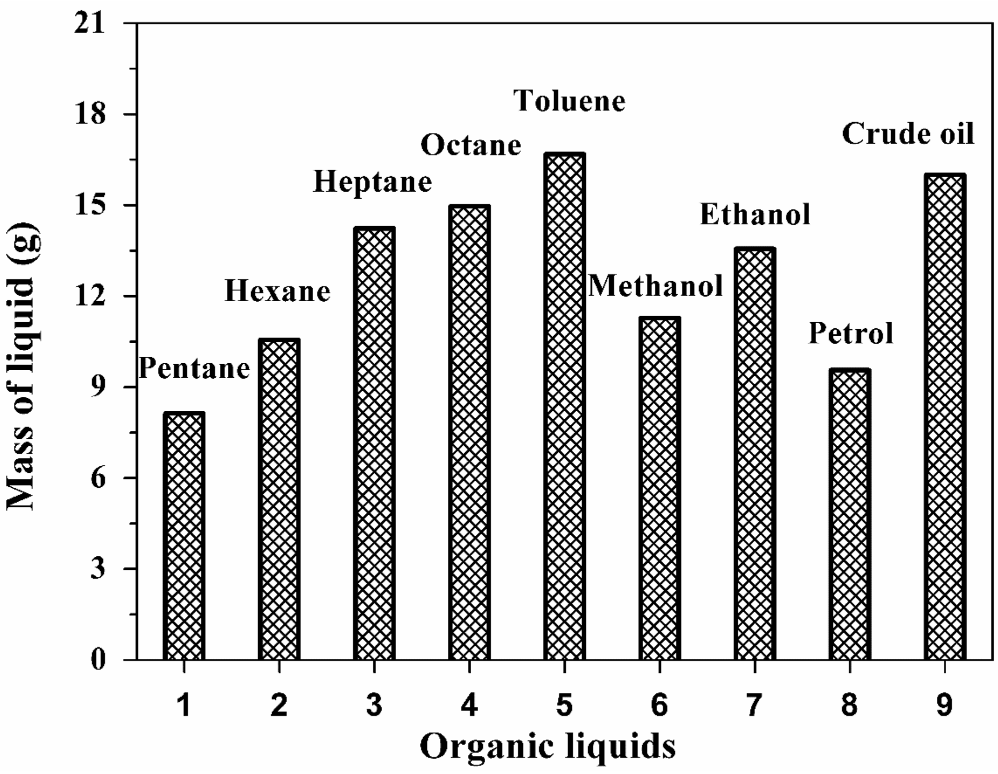

Figure S5. Mass of various organic liquids absorbed by unit mass (1 g) of the polyHIPE-silica composite sample (TPM1).

Video description

Video demonstrates the recovery of oil by applying mechanical force (by hand) from elastic silica-polyHIPE covalently bonded material (oil is absorbed as shown in Figure 5). The sample weight was noted after oil absorption; then, sample TPM 1 was compressed for oil recovery as shown in the video. After release sample regains its original shape and size within 10 sec as observed in the video. The sample was weighed to compare the weight of the sample before oil absorption and after oil recovery. 1 g of TPM1 sample absorbed 12 g oil and after recovery, 11 g oil was obtained in the first cycle. The oil absorption followed by compression and release was repeated 10 times; the material showed similar performance even after the 10 cycles. Hence, the proposed scheme is

proven highly effective from an application point of view because the material maintains its properties due to covalent bonding between them.
